# Glenoid Component Migration in Total Shoulder Arthroplasty: A Case Report

**DOI:** 10.5334/jbsr.3862

**Published:** 2025-07-17

**Authors:** Thomas Saliba, Sanjiva Pather

**Affiliations:** 1 Hôpital de Braine L’Alleud, Rue Wayez 35, 1420 Braine-l’Alleud, Belgium

**Keywords:** Total shoulder arthroplasty, pain, glenoid component, migration, complication

## Abstract

Glenoid component migration is a serious complication in total shoulder arthroplasty, causing 32% of failures and 7% of revisions. Detecting polyethylene loosening is challenging due to its radiolucency. We report a case of a 72-year-old woman with shoulder pain three years post-arthroplasty. Initial radiographs were unremarkable, but CT-arthrography revealed a displaced polyethylene spacer, rotated 90°, thus causing a mechanical block. Retrospective analysis of the X-rays showed the absence of the spacer in the expected position. Surgery restored functionality. This case highlights the diagnostic challenges of radiotransparent components with possible migration in unexplained post-arthroplasty shoulder pain.

*Teaching point:* Verify glenoid component positioning in shoulder arthroplasties.

## Introduction

Loosening of the glenoid component in total shoulder arthroplasties (TSA) is responsible for 32% of failures and 7% of revisions [1]. There is also a relatively high rate of radiological glenoid component loosening, with one study reporting that 31% of patients over 80 years with a follow‑up of at least 3 years had radiological signs of loosening of the glenoid component [2]. Spotting such failures in rare forms of displacement may be challenging. In cases where the glenoid component is significantly displaced, it can be challenging to identify. Particular attention has to be paid to atypical areas and subtle anomalies to piece together what happened to the prosthesis. This is particularly difficult as the glenoid component is radiotransparent, except for small radiopaque markers, which can be partially or completely masked by overlaying dense structures or being angulated and thus become largely invisible.

## Case Report

**Figure 1:**
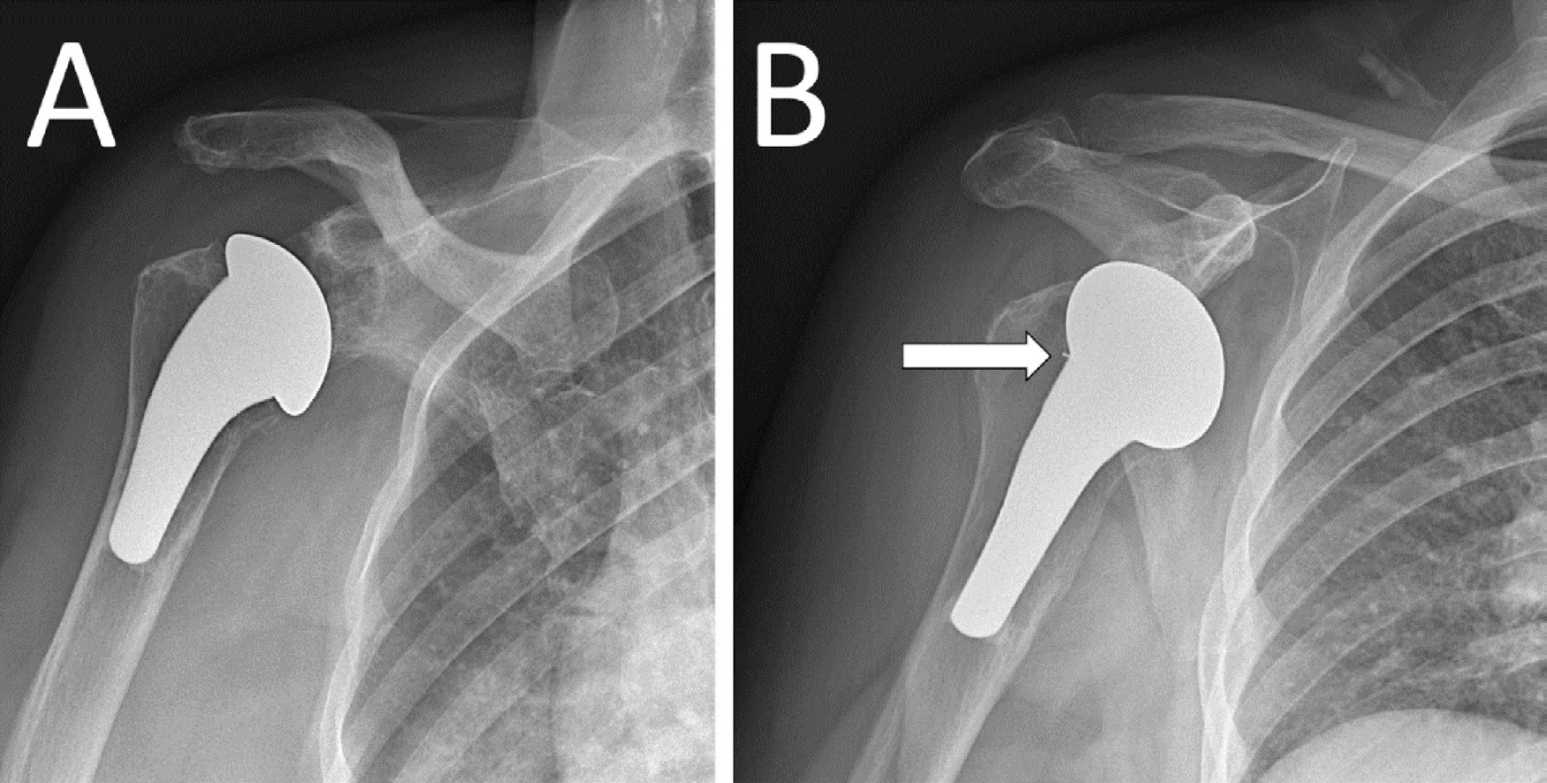
Anteroposterior **(A)** and internal rotation **(B)** X‑rays of the right shoulder. The radiopaque marker (arrow) is partially visible at the junction of the head and stem of the humeral component of the arthroplasty material, but only on the internal rotation incidence.

**Figure 2:**
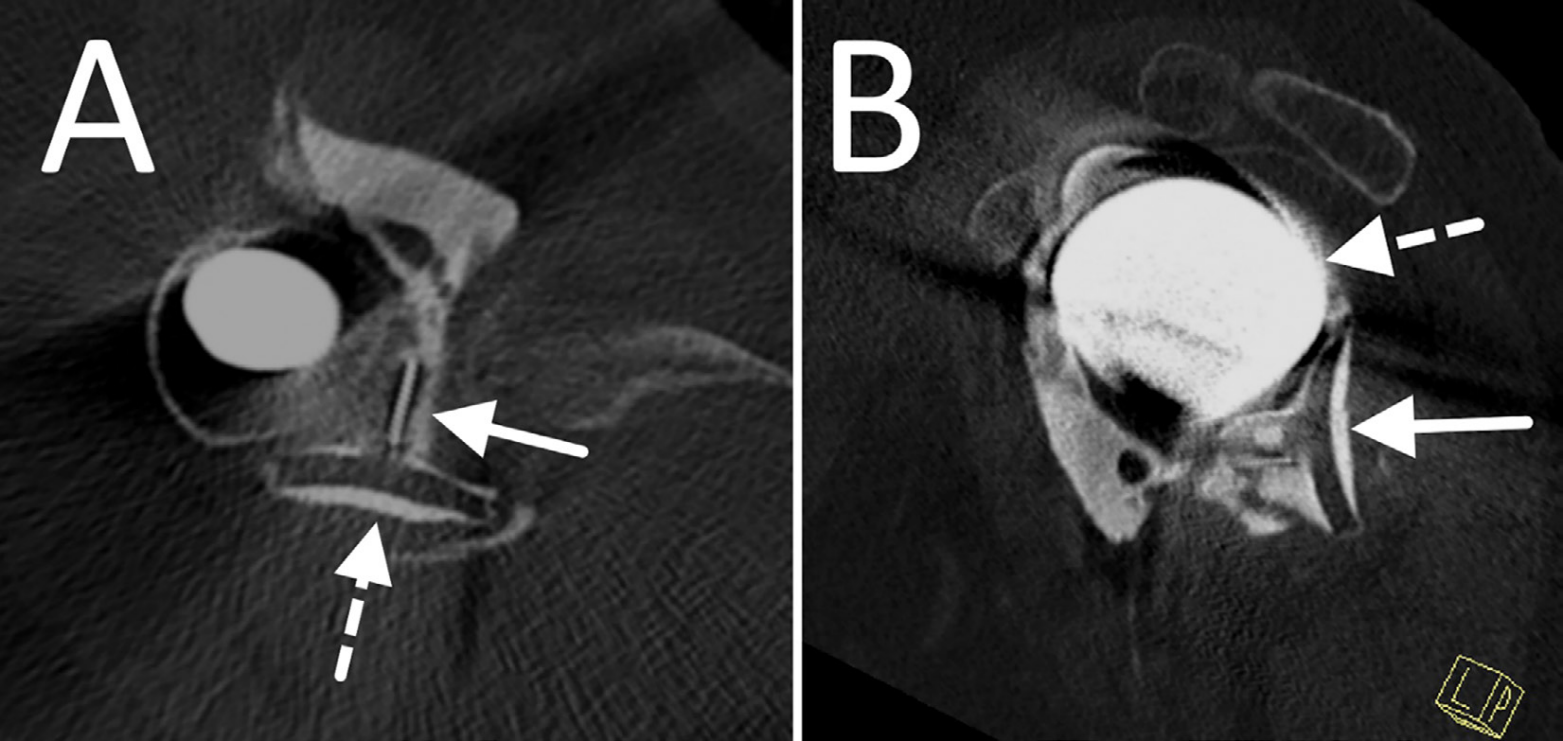
Axial **(A)** and sagittal **(B)** images of a CT‑arthrography of the shoulder. The axial image shows the glenoid component (dotted arrow) and the radiopaque marker (solid arrow) within the posterior recess of the shoulder. The sagittal image shows that the glenoid component (solid arrow) is located beneath the head of the humeral component (dotted arrow) and is facing backwards.

A 72‑year‑old woman presented with a history of right shoulder pain. She had an anatomical TSA three years earlier and recently complained of shoulder pain. A shoulder X‑ray was performed (Figure 1) and was reported as normal. As the pain did not resolve, a shoulder CT‑arthrography (Figure 2) revealed that the polyethylene spacer had moved out of the glenoid fossa and turned 90°, perpendicular to the shoulder with the concave cup facing anteriorly, thus inhibiting shoulder movement and causing pain. Retrospective analysis of the X‑ray showed the opaque marker of the polyethylene partially overlying the cortex of the humeral shaft and evidence of bone resorption around the glenoid fossa. After surgical intervention to return the spacer to its correct position, the functionality recovered well.

## Discussion

Failure of the polyethylene glenoid component is a relatively common complication [3]. This often causes pain, functional loss and a feeling of ‘clunking’ within the shoulder [3]. As the physiopathology of the component failure is poorly understood, it is difficult to take steps to prevent it [3]. However, edge‑loading, retroversion of the component and rotator cuff deficiencies are hypothesized to play a role [4].

In cases of failure and migration of the glenoid component, the distance travelled is generally limited, with few reports of complete and distant migration [5].

When verifying the glenoid component using standard radiography, one must rely on the radiopaque marker, generally found in the central keel or peg, to infer the position of the material and see that it does not move over time [4]. Radiographic signs of loosening include progressively increasing radiolucent lines around the glenoid component, which become significant at 1.5 mm, cement fragmentation and component migration or tilt [4].

CT is more sensitive to detect glenoid component failure due to better visualization of radiolucent lines and peri‑prosthetic osteolysis [4].

Detecting this anomaly may have been hindered by inattentional blindness. This phenomenon occurs when radiologists overlook significant but unexpected abnormalities while focusing on other, more subtle issues [6].

## Conclusion

In conclusion, due to the relatively high rate of failure of TSAs, it is essential to treat each case in which a patient presents with pain with a high degree of suspicion. As the glenoid component is the most common point of failure, it is important to establish if the patient has undergone a hemiarthroplasty or full arthroplasty. In cases of symptomatic TSA, careful examination of the glenoid component is required to report complications such as migration, as the small radiopaque marker clue for approving the position may be obscured by overlying structures in cases of gross migration.
